# Basics of advanced therapy medicinal product development in academic pharma and the role of a GMP simulation unit

**DOI:** 10.1016/j.iotech.2023.100411

**Published:** 2023-10-14

**Authors:** I. Johanna, A. Daudeij, F. Devina, C. Nijenhuis, B. Nuijen, B. Romberg, C. de Haar, J. Haanen, H. Dolstra, E. Bremer, Z. Sebestyen, T. Straetemans, I. Jedema, J. Kuball

**Affiliations:** 1 Department of Hematology, University Medical Center Utrecht, Utrecht; 2 Center for Translational Immunology, University Medical Center Utrecht, Utrecht; 3 Department of Pharmacy & Pharmacology, Netherlands Cancer Institute, Amsterdam; 4 Department of Pharmacy, University Medical Center Utrecht, Utrecht; 5 Department of Medical Oncology, Netherlands Cancer Institute, Amsterdam; 6 Division of Molecular Oncology and Immunology, Netherlands Cancer Institute, Amsterdam; 7 Laboratory of Hematology, Department of Laboratory Medicine, Radboud University Medical Center, Nijmegen; 8 Department of Hematology, University Medical Center Groningen, University of Groningen, Groningen, The Netherlands

**Keywords:** cellular therapy, ATMP, GMP, process development, clinical translation

## Abstract

Following successes of authorized chimeric antigen receptor T-cell products being commercially marketed in the United States and European Union, product development of T-cell-based cancer immunotherapy consisting of cell-based advanced therapy medicinal products (ATMPs) has gained further momentum. Due to their complex characteristics, pharmacological properties of living cell products are, in contrast to classical biological drugs such as small molecules, more difficult to define. Despite the availability of many new advanced technologies that facilitate ATMP manufacturing, translation from research-grade to clinical-grade manufacturing in accordance with Good Manufacturing Practices (cGMP) needs a thorough product development process in order to maintain the same product characteristics and activity of the therapeutic product after full-scale clinical GMP production as originally developed within a research setting. The same holds true for transferring a fully developed GMP-grade production process between different GMP facilities. Such product development from the research to GMP-grade manufacturing and technology transfer processes of established GMP-compliant procedures between facilities are challenging. In this review, we highlight some of the main obstacles related to the product development, manufacturing process, and product analysis, as well as how these hinder rapid access to ATMPs. We elaborate on the role of academia, also referred to as ‘academic pharma’, and the added value of GMP production and GMP simulation facilities to keep innovation moving by reducing the development time and to keep final production costs reasonable.

## Highlights

• Complex pharmacological characteristics of ATMPs pose a challenge for translation.
• Product development process and critical product quality attributes need attention.
• Standardization of manufacturing processes and quality-control methods is crucial.
• Academic GMP simulation units aim to bridge the knowledge gap in ATMP development.
• GMP simulation units support faster bench-to-bedside translation of new ATMP leads.

## Introduction

Favorable clinical outcomes resulted in the implementation of an increasing number of living advanced therapy medicinal products (ATMPs) as the standard of care for cancer patients worldwide, including T-cell-based therapies. Many more clinical studies have been initiated to increase efficacy of a next-generation ATMP in so-far established indications as well as to broaden indications towards many other malignancies.^1^, ^2^, ^3^ Within this context, novel strategies are explored to optimize the complex interplay between immune cells and cancer cells, and to improve access of engineered immune cells to the tumor microenvironment in order to increase efficacy without toxicity.^4^ In addition, ATMPs are still most frequently developed in a patient-specific manner, with individual differences, e.g. in starting material composition, influencing final product characteristics, and quality attributes, resulting in complex manufacturing and quality-control processes compared to conventional biological products.^5^ Thus, distinct regulatory guidelines and product assessments of ATMPs for clinical translation to comply with the current Good Manufacturing Practices (cGMP) are required and have been taken into consideration by developers, pharmacists, and regulatory bodies, including technology transfer processes involved within a GMP-grade facility and from one to another facility.^6^^,^^7^

To accelerate clinical translation of ATMPs, close collaboration between academia, industries, and regulatory bodies is needed to ensure better access to sustainable advanced therapy products. Several programs have been initiated to facilitate collaboration between multiple stakeholders to facilitate ATMP development, such as PRIority MEdicines (PRIME) and Accelerating Clinical Trials in the European Union (ACT EU) initiated by the European Medicines Agency (EMA). Within this context, we highlight the potential role of academic GMP production and simulation units. An academic pharma production unit produces fully developed ATMPs for clinical trials or standard of care and could also serve as a satellite production center for pharmaceutical industry productions, which could produce ATMP potentially faster and at lower costs. An academic GMP simulation unit is a separate facility that creates a pre-GMP environment and consists of a core team of translational scientists and GMP production experts (including pharmacists) with specific focus on bridging the gap between preclinical research and GMP production of ATMPs for clinical application as also emphasized by others.^8^ GMP simulation units can assist in the product development stage from preclinical innovation and process implementation towards a GMP production facility, which entails setting up and executing initial qualification and validation activities related to the drug product in development. GMP simulation units can be also involved in harmonization efforts of production processes and product characteristics as well as supporting product assessment towards market authorization to assist all stakeholders involved during development and approval of ATMPs.

## Basic principles of product development from bench to a GMP-compliant procedure

**Figure 1:**
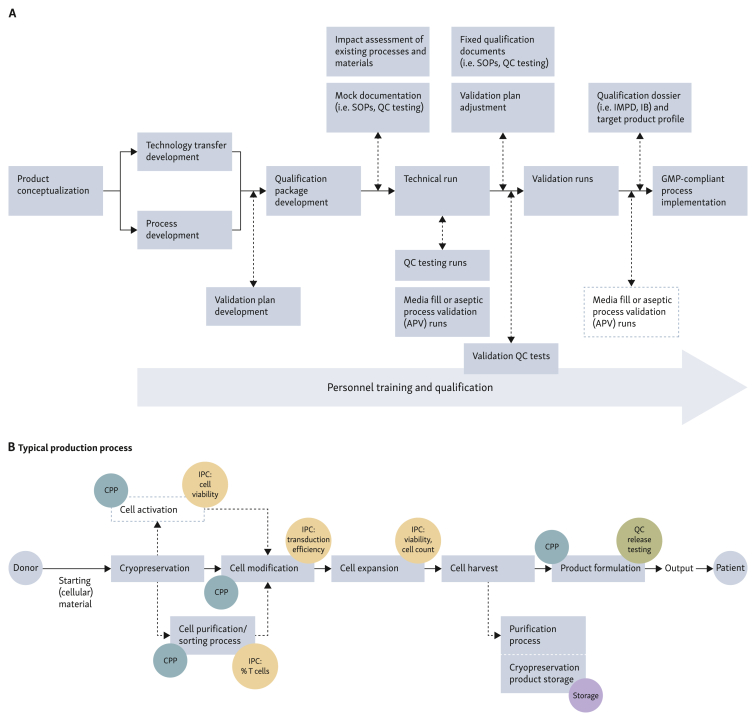
**QbD approach at the earliest phase of ATMP development.** (A) Workflow of a GMP simulation unit. Indicated are different steps of a QbD strategy which is carried out within a GMP simulation unit to facilitate a rapid path of innovation towards a GMP-compliant ATMP. (B) Typical production process of T-cell-based therapeutic products. Highlighted are CPP and potential IPCs. ATMP, advanced therapy medicinal product; CPP, critical process parameters; GMP, Good Manufacturing Practices; IB, Investigator's Brochure; IMPD, Investigational Medicinal Product Dossier; IPC, in-process control; QbD, quality by design; QC, quality control; SOP, standard operating procedure.

A proper product and process conceptualization from a research-grade product to a GMP-compliant product is needed to gain knowledge about product feasibility and good estimation of the production costs per treatment. In parallel, a careful market analysis in terms of medical need, intellectual property, and potential financial benefit is needed, given the costs associated with preclinical and clinical development aiming to approve novel ATMPs. In our experience, this process can take nearly a decade from a first-lead description^9^ until the decision is made to start a clinical trial either from academia^3^ or by companies.^10^ To tackle challenges identified in the context of drug development and manufacturing failures, implementation of a quality-by-design (QbD) framework, a systematic approach to drug product development based on scientific knowledge and quality risk assessment pertaining to manufacturing processes at the earliest phase of ATMP development is important (Figure 1). The QbD strategy is largely unknown to many researchers developing completely new ATMPs and therefore the concept of QbD is described in more detail here, to raise an understanding of the complexity of later ATMP development. QbD includes an early description of the quality target product profile (QTPP). As per the quality guideline of the International Council for Harmonisation (ICH Q8) for Pharmaceutical Development,^11^^,^^12^ QTPP is the prospective summary of the quality characteristics of a drug product, ensuring the desired quality, efficacy, and safety of the drug product. QTPP is a tool to identify the critical quality attributes (CQA) early in the product development stage. CQA encompass physical, chemical, and biological properties of the drug product that should be within appropriate limits, ranges, or distribution, to ensure the desired product quality according to relevant regulatory guidelines and prior product knowledge or similar comparator products.^13^ Based on QTPP and CQA, critical process parameters (CPP) and other key process parameters need to be established to determine which process steps are crucial and need to be controlled with selected analytical methods. In addition, in-process controls (IPCs) need to be defined such as e.g. efficiency of transduction of T cells or expansion rate of T cells and monitored to assess whether they are truly relevant and validated for process consistency as illustrated in Table 1.^14^ CPP are determined using risk priority numbers to assess any potential failure during the production process based on their severity, occurrence, and detection level.^15^ The determination of CQA and CPP allows a better prediction of the final drug product outcome. By adopting a QbD framework during the development of a GMP process, modifications after a clinical study has started can be prevented—unless strictly necessary—and the risk of late-stage product development failure is minimized. Nonetheless, implementation of a QbD approach for cell-based therapeutics remains challenging due to the personalized nature of advanced therapies, lack of understanding of complete mode of action, and its impact on product quality attributes, and limited technological advancement for process analytical methods.^16^ Better understanding of quantitative molecular (e.g. percentages of a protein expressed on a cell membrane) and cellular characteristics (e.g. phenotype of cells) as well as their relation to final product quality is fundamental in the full implementation of QbD framework for ATMP development.

**Table 1:** Example of assessment exercise in determining QTPP, CQA, CPP, and IPC in ATMP production process^11^^,^^12^

QTPP element	Target	CQA (yes/no)	Justification	CPP	IPC
Dosage form	CAR T cells diluted in formulation buffer	No	Not applicable	Not applicable	Not applicable
Appearance by visual observation	Clear to slightly turbid solution with possible visible cells agglomerate	Yes	Solution clarity can be used as immediate sign of foreign particle contamination	Media exchange	Not applicable
Identity	Transduction efficiency	Yes	It affects the consistency of the dosage given	Transduction process	- Day 5 - Day 12
	Immune phenotype	Yes	To ensure the cells are transduced properly into CAR T cells	Transduction process	- Day 5 - Day 12
	Cell morphology	No	The cell morphology may be indicating cell health and is affected by the transduction rate but is not a direct indication of the quality of the treatment	Culture process	- Day 5 - Day 12
	Transgene copy number per cell	No	The transduction rate shall not change after the process is completed	Transduction process	Not applicable
Potency	Cytolytic activity^26^	Yes	To confirm consistency, stability, and quality between lots	Not applicable	Not applicable
Purity	Viability	Yes	It may affect the dosage given	- Donor cells - Culture process - Media exchange	- Day 1 - Day 5 - Day 12
	% T cells	No	It affects the product yield	Donor cells	Cell surface marker
	% CAR^+^ cells in viable CD3^+^ cells	Yes	It may affect the dosage given	Transduction process	- Day 5 - Day 12
Impurities	% B cells and monocytes % CD45^−^ tumor cells	Yes	It may affect patient safety and the dosage given	Donor cells	Cell surface marker
	Ancillary materials (e.g. residual activation beads)	No	To confirm process consistency	- Culture process - Media exchange	Not applicable
Safety^41^	Sterility	Yes	It affects patient safety	- Donor cells - Culture process - Media exchange	Not applicable
	Mycoplasma	Yes	It affects patient safety		
	Endotoxin	Yes	It affects patient safety		
	Integration site analysis	Yes	It addresses the risk deriving from insertional mutagenesis	Not applicable	Not applicable
	Vector copy number per cell	Yes	It affects patient safety	Transduction process	Not applicable
	Replication competent lentivirus/retrovirus	Yes	It affects patient safety	Transduction process	Not applicable
	Off-target effects (i.e. for gene-edited ATMPs)	Yes	It affects patient safety	Not applicable	Not applicable
Container closure^43^	Support product stability during transportation and storage	No	To be proven by stability study Part of container closure release testing	Not applicable	Not applicable
Pharmacokinetics^43^	Persistency and functionality of drug product versus product design	No	To be proven in animal and human studies to be justified	Not applicable	Not applicable
Efficacy^43^	Correlation between potency and efficacy	No	To be proven in human efficacy data	Not applicable	Not applicable

ATMP, advanced therapy medicinal product; CAR, chimeric antigen receptor; CPP, critical process parameters; CQA, critical quality attributes; IPC, in-process control; QTPP, quality target product profile.

A product development strategy provides technical gap analysis related to the product and manufacturing process and provide sufficient historical nonclinical data and prior knowledge from comparator products to support further product development. Based on the gap analysis and product knowledge, a product development plan is put in place to establish an optimal and robust manufacturing process to achieve maximum drug product efficiency without compromising product quality.^17^ This product development plan should be in place before process translation to a GMP-complaint process, more specifically before or during early product development. This covers multiple process elements, including product scalability that includes the possibility to scale-up, scale-down, or scale-out the production process to cater the intended clinical application. This step is particularly important for cell-based therapeutics where cellular behavior significantly influences expansion capacity, especially with different culturing systems. We conclude that a well-established product development strategy has to be in place to effectively convert the research laboratory protocol to a GMP-compliant manufacturing process based on product, process, and analytical method knowledge.

## The abc of analytical QC methods for ATMP manufacturing

To ensure product quality and process reproducibility, product characterization and quality control (QC) are particularly important for cell-based ATMPs as these ‘living drugs’ are inherently variable with regard to cell composition, proliferation ability, viability and antigen recognition capabilities due to their frequently personalized nature or the fact that even for third-party products different donors are usually needed to produce multiple batches to treat larger patient populations. Analytical assays have to be carried out for every production batch as part of product release and established based on product quality attributes. Validation of analytical QC testing is carried out to demonstrate that the assay is suitable, precise, and reproducible throughout all production batches. According to ICH Q6B and aligned with European Pharmacopeia (Ph. Eur.) reference methods, the typical QC release assays for biological products mainly include: (i) product identity, (ii) purity and impurities, (iii) product quantity and/or product potency, and (iv) safety.

**(i) Product identity** should be characterized, from a pragmatic point of view, as simple as possible and based on unique aspects of the ‘molecular structures’ or other specific properties of the drug product. It ensures that the drug product contains the therapeutic cell subset, which is a major challenge for ATMPs given the nature of the living drug product which has a high diversity in cellular composition with many active and non-active ingredients, as reported for expanded tumor-infiltrating lymphocytes in which ∼30% are tumor reactive^18^^,^^19^ and engineered T cells with transduction efficiency ranging between 20% and 70%.^20^^,^^21^ Means to enrich engineered immune cells are therefore investigated.^22^ To determine product identity of cellular therapy products, surrogate markers associated with functional activity are used, such as measurement of specific cell surface markers for phenotypic signature using flow cytometry-based methods or assessment of transgene expression by measuring DNA copy number using quantitative PCR (qPCR) method.^23^ The current drawback of flow cytometry-based characterization of ATMPs is that, despite the availability of advanced machines that can assess >40 different parameters, it still provides limited characterization considering the diversity of the product. These methods are also restricted by the availability of already-developed reagents and markers, and can be costly.^24^ To determine a more comprehensive product identity, single-cell RNA sequencing has been used to describe the transcriptomic signature and potency of individual cells and products.^25^ This approach is not currently feasible for immediate-release tests but in selective cases could help in discussions with authorities to justify major changes in production workflows and support validity of less complex assays.

**(ii) Product purity and impurities** of cell-based ATMPs are difficult to determine and mostly requires a combination of methods. Product purity determines the rate of desired therapeutic cell subsets in the presence of other cell types. In this context, cell viability and cell numbers per target dose can be used as product purity parameters and associated potency based on the assumed mechanism of action^26^ to monitor ATMP-manufacturing processes and is also often selected as one of the IPCs for critical steps. Various analytical methods are used for cell counting and viability, including manual trypan blue exclusion,^27^ automatic cell counters,^28^^,^^29^ flow cytometry-based methods using live/dead cell markers or Trucount beads,^30^ and built-in fluidic-based system based on volumetric method that enable precise automated cell counting in a single-platform flow cytometry.^31^ However, real-time analysis of cellular products remains difficult, especially for adherent cells. Recently, a white light spectroscopy system that uses the optical absorption method was developed to allow for real-time cell counting.^32^ Moreover, process- or product-related impurities, including undesired cell types (i.e. B cells, monocytes, and CD45^−^ tumor cells), residual ancillary materials (i.e. activation beads, viral vectors, cytokines, and serum used during production), non-cell particulates (e.g. plastic fragments, residual microfibers), or leachable substances (compounds that make their way into the product mainly through normal contact with polymer-based material originated either from a container-closer system or device component) have to be sufficiently removed as they may alter efficacy and safety profiles of the drug product.^33^

**(iii) Potency assay** is a quantitative measure of biological activity based on the intrinsic functionality of drug products related to their known mode of action. These assays do not directly measure clinical efficacy, instead they are used to measure relevant biological activity related to the intended therapeutic effect of the drug product and to evaluate the product consistency in the case of process changes. Currently, simple potency assays are included as part of the product release testing and carried out for each production lot, although they are not required to be validated before early-phase clinical trials.^34^ The difficulty in development of such an *in vitro* potency assay for ATMPs is the fact that this assay may not entirely represent the multiple factors that influence biological activity of the drug product once administered to the patient, and thus most likely does not accurately predict *in vivo* efficacy. Furthermore, commonly used potency assays may be lacking a direct correlation of short-term lytic activity measured in the assay with long-term effect of drug products in patients and the two-dimensional assay setup does not signify the complexity of the tumor microenvironment to the overall efficacy profile of the drug product. Thus, careful assessment of adequate potency assays remains crucial. The development of multiplex or overarching assays is preferable to better characterize the potency of drug products, such as the abovementioned single-cell transcriptomic analysis as a surrogate potency assay. On the other hand, including an advanced potency assay during release testing may be difficult to standardize, and thus do not allow reliable reproducibility, and are also time-consuming and expensive. Due to the complexity of the mode of action of ATMPs, no standardized potency assay has been established to date.

**(iv) Safety** in biotechnological products mainly ensures that the drug product is free of microbiological contamination, in addition to the unwanted potentially harmful impurities mentioned before, such as bacteria or fungi, mycoplasma, and endotoxin, as final product sterilization is not possible for living cellular products. Sterility testing takes ∼10-21 days of culture period depending on the methods used and thus the results may not be available before product release and administration to the patient if a fresh drug product is to be administered. Although not favorable, the administration of ATMPs directly after production is applicable when the drug product cannot be stored for a longer period either because of the product stability or the patient demand. Several automated systems for rapid sterility testing, including BacT/ALERT and BACTEC in accordance with Ph. Eur. 2.6.27 reference methods,^35^ are usually accepted to have preliminary results after 4-5 days and can be used to justify conditional release of the investigational drug product before infusion into the patient. Development of shorter culture systems for immediate product release would be beneficial for ATMPs to ensure product safety in a timely manner before infusion.^13^ Safety parameters associated with the widely used viral-based gene transfer and gene editing tools should also be considered in the product safety assessment of cell-based gene therapy products, including information regarding residual vector copy numbers, target integration site profile of the introduced transgene, and absence of a replication competent viral vector in the final drug product.^24^

## Towards novel analytical QC methods

Given the complexity of ATMPs, standardization of existing and novel analytical QC methods is important in GMP-compliant ATMP manufacturing. However, very few publications are available with regard to analytical method development and relevant approaches chosen to validate such tests, especially for product identity, purity, and potency. This leads to poor consensus on which parameters are required to determine quality and functionality of the drug product and also to inconsistencies between different developers that do not use the same parameters. Timing of donor availability, donor variability, and cellular heterogeneity of ATMPs also add variability in sample representation during analytical method development. In addition, detailed scientific knowledge of the mode of action and availability of reference standards are needed for analytical method development and validation. Proposed guidelines for analytical methods used for cellular products, including Ph. Eur., ICH Q2(R1) and ICH14, are mainly focusing on microbiological controls, while specific guidelines for ATMPs remain underrepresented. Thus, analytical method development for ATMPs remains challenging. A progressive standardization approach to establish well-validated analytical methods during product development that could be shared across ATMP-manufacturing facilities globally would help clinical translation. By streamlining sets of QC assays used for different cellular therapy products, we could provide a common framework and indicate the minimum types of assays that are required for characterizing CQAs throughout manufacturing processes, which then could be used for any future studies. Within this context, it will be interesting to explore whether characterization of engineered cellular products by their transcriptomic landscape, albeit the transcriptomic signature might not always reflect protein expression,^36^ could allow a better fine-tuning to establish truly relevant potency assays that better predict clinical efficacy.^25^ In addition, such a strategy would also allow better assessment of the impact of major production changes to product potency and open an avenue towards assessing the power of biosimilars of ATMPs without the need for larger clinical trials. Lessons learned from molecular analyses of engineered T cells from long-term survivors might open a new avenue towards novel QC methods.^37^

## Automated procedures: more quality with more choices?

As part of a product development, early adaptation of mostly open-system and manual production processes into automated and closed manufacturing platforms is favorable because it will significantly reduce the risk of microbiological contaminations and may reduce variability. This can be also done modularly by integrating automation for, e.g. individual unit operations.^38^ Advantages of automation include ensuring manufacturing reproducibility and robustness of the process. Automated technologies are favorable for GMP manufacturing to facilitate closed-system culture for ATMPs, including scalable bioreactors for cell expansion and automated cell-processing devices. The use of automated cell-processing platforms and robotic devices in ATMP manufacturing will significantly reduce the need for manual manipulation, while retaining the necessary oversight by well-trained personnel, and allowing process standardization by eliminating personnel-related variability.^39^ Considering the nature of the living drugs, ATMP production requires precise production planning as failure in one step of the manufacturing process, especially relating to a single device, will significantly impact production flow. The main challenge with the use of automated platforms and their related software programs is the poor cross-compatibility between devices due to innovation gaps and increased dependency on a specific supplier for that particular automation platform.^38^ Consequently, proper assessment of a possible interchangeability between different automated platforms, different processes, or different units of operations within the same process where the same product type can be produced without significantly altering product characteristics, carried out in the early stage of product development, will greatly aid in their clinical application. Such standardization will allow an efficient technology transfer process between manufacturing facilities and developing of modular devices and e.g. is part of the Dutch Innovation program NXTGEN-HIGHTECH (https://nxtgenhightech.nl).

## How to learn from failures of ATMP production?

Even after ATMP production processes are established and clinical trials are running or products are approved, production failures are observed in clinical trials and for approved products, in particular when using patient-derived starting material.^40^ However, most of the time, detailed data on production failures and thorough analyses are not elaborated in the published database.^24^ Only few studies investigated what factors or cell characteristics influenced the production failures, which hinders necessary improvement of production processes for products with similar characteristics and production processes. Such corrective feedback loops are actually crucial to optimize future ATMP production, where eventually we can even predict factors that determine production failures and successes, e.g. cell functionality of the starting materials could influence the overall manufacturing process development and product characteristics.^25^

## Overcoming challenges in clinical translation of atmp and the role of academic GMP simulation teams and units in product development

Most frequently important details of manufacturing processes are not publicly shared. This is one of the reasons for the lack of standardization across production processes and analytical QC methods in ATMP manufacturing, which are significant obstacles for clinical translation. Various studies are consequently carried out with multiplicity of approaches for the same product type. This leads to lack of comparability between different strategies and poor or publicly not available product characteristics. The knowledge gap of academic researchers who have a promising therapeutic concept well tested in a research laboratory environment, but are not equipped with the necessary expertise in the requirements for clinical translation, is one main obstacle.^41^ The inclusion of clinical trial, registration, and GMP production experts at an early stage is crucial, as trials should always have market approval in mind and the product design could impact production processes (e.g. frozen versus fresh products, centralized versus de-centralized production). Such a multidisciplinary ATMP knowledge team can serve not only for the development of ATMPs in the field of oncology, but also other fields like regenerative medicine. Throughout the product development lifecycle, this multidisciplinary team will not only be working on developing a GMP-compliant production process, but also improving QC methods, bridging the length, and challenging analysis without hindering the production process itself.

**Figure 2:**
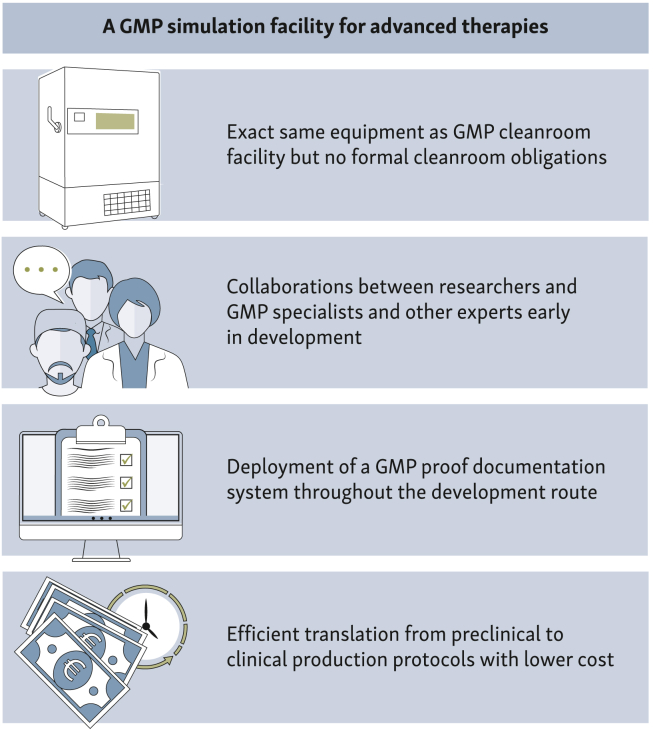
**Essentials of a GMP simulation facility.** GMP, Good Manufacturing Practices.

**Figure 3:**
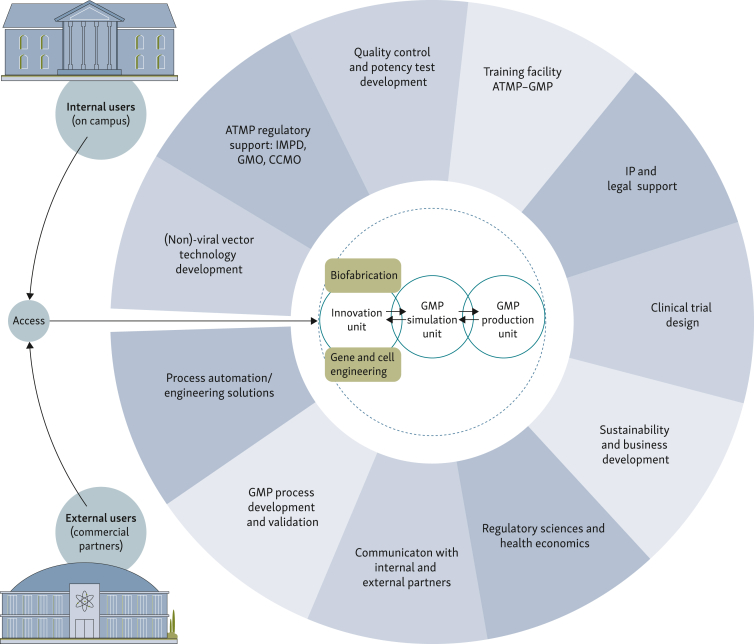
**Setup of the Innovation Center for Advanced Therapies (ICAT).** Indicated are different stakeholders and positioning of a GMP simulation unit within an innovation environment. Internal users: all users within the campus of this facility. External users: other academic or commercial partners. ATMP, advanced therapy medicinal product; CCMO, Centrale Commisie Mensgebonden Onderzoek (English: Central Committee on Research Involving Human Subjects); GMO, Genetically Modified Organism; GMP, Good Manufacturing Practices; IMPD, Investigational Medicinal Product Dossier; IP, intellectual property.

An interesting option, though possibly not feasible at all development and production facilities, is the building of a separate GMP simulation facility that fosters close collaboration between researchers and product developers, GMP specialists, and other experts early in the product development pipeline (Figure 2), as suggested also by others.^8^ In this integrated development facility, GMP-grade equipment and methodologies are used similar to a GMP cleanroom facility, with the main objective of gaining better insight into what is needed for the process to meet all quality requirements. A process validation-dedicated cleanroom embedded within the GMP production facilities will be used during the final phase of product development to establish and validate GMP production processes directly in the GMP-grade environment, in order to avoid the hurdles of additional process transfer. That being said, the product development process is continued throughout the clinical phase itself, during which the GMP simulation and GMP production teams work together as one stream to facilitate faster clinical translation and process improvement when necessary. In particular, when developing modular modes with different machines, such flexibility can be important. Thus, GMP simulation units may facilitate efficient and cost-effective ATMP development, validation, process optimization, and implementation from preclinical to clinical production protocols, as reported by other centers. Although the GMP simulation facility is a separate infrastructure meant to bridge preclinical development and GMP-complaint production, both GMP simulation and GMP production facilities will join forces to support faster translation of new product leads from research to the clinics. In Utrecht, a GMP simulation unit that specifically focuses on product development of ATMPs is currently being established, embedded within the Innovation Center for Advanced Therapy (ICAT) as a collaborative effort of research institutes in Utrecht Science Park, The Netherlands. This facility aims to bridge the gap between preclinical development to first-in-men clinical studies and accelerate bench-to-bedside clinical translation. Close interaction between academia within universities and research institutes, and partnerships with biopharma industries are essential for success (Figure 3).

## Expanding the network

To increase accessibility of cellular therapy, harmonization effort across multicenter academic cell therapy facilities is essential. Development of a centralized (academic) platform to allow data exchange pertaining to ATMP production is therefore important. In this context, we initiated a **D**utch platform for cancer-specific **A**TMP **Re**search to ensure harmonized development, clinical testing, and sustainable patient access (DARE-NL; www.dare-nl.nl) project across academic centers, to build a national transdisciplinary multi-stakeholder infrastructure for rapid bench-to-bedside ATMP translation, comprising scientific knowledge hub, biological and technology platforms, implementation, and patient outreach program. Firstly, DARE-NL will establish an ATMP knowledge framework by building an Information and Communication Technology infrastructure that supports data and knowledge exchange from different academic centers and enables harmonization of GMP development and production processes, including the implementation of standardized QC assays in ATMP production. Data harmonization of production processes, quality management system, validation, and analytical QC methods strategies will reduce product variability, increase reproducibility, and enable site-to-site comparison.^42^ Secondly, DARE-NL aims to create a biologics and technology hub that supports development of innovative technologies that support ATMP production, such as availability of GMP-grade materials and viral vectors for academic use and future outlook to new technological advancement applicable for ATMP manufacturing. Using the DARE-NL platform, we aim to strengthen the connection towards policy makers in The Netherlands and across EU pertaining to regulatory issues, health technology assessment, market authorization, and reimbursement program, as well as a patient outreach program to improve patient access. A next level of innovation and harmonization is offered within the recently funded Oncode-PACT project (https://www.oncode.nl/oncode-pact). Such harmonization effort remains a challenging process, but it is essential in ATMP manufacturing.

## Conclusion

ATMPs require a specialized advanced technology translation from research-grade processes to GMP-compliant production processes. To address challenges in clinical translation of ATMPs in academic pharma settings, harmonization and collaborative efforts between multidisciplinary stakeholders must be established from an early stage of product development to streamline the ATMP network and accelerate their clinical translation. Dedicated GMP simulation units might be one solution to bridge this knowledge gap through dedicated personnel trained in research and also GMP needs, regulation, and facilities.

## Disclosure

**JH**: Grants to institute from BMS, Novartis, Amgen, Asher Bio, BioNTech; consulting fees from Achilles Tx, BioNTech, BMS, Gadeta, GSK, Iovance, Instil Bio, Immunocore, MSD, Merck Serono, Molecular Partners, Novartis, PokeAcel, Pfizer, Roche, Sanofi, T-Knife to institute; consulting fees from Neogene Tx, CureVac, Scenic to self; support for attending meetings and/or travel: BioNTech; leadership role: CCMO, ESMO Council, ESMO IOTECH Editor-in-Chief; stock options: Neogene Tx; **HD**: Inventor patent: Improved method for ex vivo expansion CD34+HSPCs into NK cells using an aryl hydrocarbon receptor antagonist (PCT/EP2016/071660; patent holder Radboudumc); EBMT treasurer and board member; **ZS**: Patent licenses to Gadeta and Miltenyi Biotech; **IJ**: Dutch Cancer Society grant 13876, DARE-NL consortium; **JK**: was Gadeta shareholder; patent licenses to Gadeta and Miltenyi Biotech; participation on a data safety monitoring board or advisory board: On CAR T studies; leadership role: EBMT Chair LRAC, Board HOVON. All other authors declare no conflicts of interest.
